# Circulating levels of soluble Fas (sCD95) are associated with risk for development of a nonresolving acute kidney injury subphenotype

**DOI:** 10.1186/s13054-017-1807-x

**Published:** 2017-08-17

**Authors:** Pavan K. Bhatraju, Cassianne Robinson-Cohen, Carmen Mikacenic, Susanna Harju-Baker, Victoria Dmyterko, Natalie S. J. Slivinski, W. Conrad Liles, Jonathan Himmelfarb, Susan R. Heckbert, Mark M. Wurfel

**Affiliations:** 1 Pulmonary and Critical Care Medicine, University of Washington, Harborview Medical Center, 325 9th Avenue, Seattle, WA 98104 USA; 2 Kidney Research Institute, Division of Nephrology, University of Washington, Seattle, WA USA; 3 University of Leeds, Leeds, UK; 4 Department of Medicine, University of Washington, Harborview Medical Center, Seattle, WA USA; 5 Department of Epidemiology, University of Washington, Seattle, WA USA

**Keywords:** Apoptosis, Acute kidney injury, Biomarkers

## Abstract

**Background:**

Critically ill patients with acute kidney injury (AKI) can be divided into two subphenotypes, resolving or nonresolving, on the basis of the trajectory of serum creatinine. It is unknown if the biology underlying these two AKI recovery patterns is different.

**Methods:**

We measured eight circulating biomarkers in plasma obtained from a cohort of patients admitted to an intensive care unit (ICU) (*n* = 1241) with systemic inflammatory response syndrome. The biomarkers were representative of several biologic processes: apoptosis (soluble Fas), inflammation (soluble tumor necrosis factor receptor 1, interleukin 6, interleukin 8) and endothelial dysfunction, (angiopoietin 1, angiopoietin 2, and soluble vascular cell adhesion molecule 1). We tested for associations between biomarker levels and AKI subphenotypes using relative risk regression accounting for multiple hypotheses with the Bonferroni correction.

**Results:**

During the first 3 days of ICU admission, 868 (70%) subjects developed AKI; 502 (40%) had a resolving subphenotype, and 366 (29%) had a nonresolving subphenotype. Hospital mortality was 12% in the resolving subphenotype and 21% in the nonresolving subphenotype. Soluble Fas was the only biomarker associated with a nonresolving subphenotype after adjustment for age, body mass index, diabetes, and Acute Physiology and Chronic Health Evaluation III score (*p* = 0.005).

**Conclusions:**

Identifying modifiable targets in the Fas-mediated pathway may lead to strategies for prevention and treatment of a clinically important form of AKI.

**Electronic supplementary material:**

The online version of this article (doi:10.1186/s13054-017-1807-x) contains supplementary material, which is available to authorized users.

## Background

Acute kidney injury (AKI) is common in the intensive care unit (ICU) and is associated with substantial morbidity and mortality [1–5]. Development of AKI occurs in response to a variety of toxic, inflammatory, and ischemic events, with the most common predisposing risk being sepsis. Biomarkers of functional (serum creatinine [SCr], blood urea nitrogen) or structural (neutrophil gelatinase-associated lipocalin, kidney injury molecule 1) kidney injury have been shown to have some utility in the early identification of AKI [6–8]. However, further exploration of the biologic pathways linked to the development of more severe forms of AKI is needed.

AKI is a heterogeneous entity, and increasing evidence shows that the Kidney Disease: Improving Global Outcomes (KDIGO) criteria for AKI severity may not adequately capture this heterogeneity [9–11]. In clinical trials, this heterogeneity may obscure treatment effects that are present only in subgroups of patients, potentially contributing to the increasing number of negative interventional trials in AKI [12]. We recently identified two AKI subphenotypes (resolving and nonresolving) on the basis of the trajectory of SCr in the first 3 days after hospital presentation [13]. A resolving trajectory, defined as a decrease in SCr of 0.3 mg/dl or 25% from the maximum, was associated with the same risk of hospital mortality as that of subjects with no AKI. A nonresolving trajectory, defined as AKI that did not meet the resolving criteria, was associated with a 60% higher risk of hospital mortality relative to patients with no AKI, even after adjusting for KDIGO stage of AKI and other potential confounders. Traditional prognostic risk factors, such as Acute Physiology and Chronic Health Evaluation III (APACHE III) score, vasopressor use, or sepsis status, did not differ across AKI subphenotypes. Prior work, primarily in animal models, has implicated different biologic processes, such as apoptosis, inflammation, and endothelial dysfunction, in the pathogenesis of AKI [14–17]. It is not yet known whether these different pathophysiologic processes might contribute to the development of a nonresolving as opposed to a resolving AKI subphenotype.

In this report, we present results of analyses based on testing of whether circulating levels of biomarkers representative of two main biologic pathways, endothelial dysfunction (angiopoietin 1 [Ang-1], angiopoietin-2 [Ang-2], angiopoietin ratio [Ang-2/Ang-1], soluble vascular cell adhesion molecule [sVCAM]) and inflammation/apoptosis (soluble Fas [sFas], soluble tumor necrosis factor receptor 1 [sTNFR-1], interleukin-6 [IL-6], and IL-8), are differentially associated with AKI subphenotypes in a cohort of ICU patients with the systemic inflammatory response syndrome (SIRS). These analyses seek to shed light on the biology of these two distinct AKI subphenotypes, resolving versus nonresolving.

## Methods

### Study design

We performed this study using previously collected data from the Harborview Medical Center cohort with systemic inflammatory response syndrome (HMC-SIRS) [18]. The HMC-SIRS cohort comprised consecutively enrolled subjects meeting criteria for SIRS [19], excluding patients with major trauma, intracranial hemorrhage, HIV infection or immunosuppression, or a current diagnosis of cancer, as previously described [20–22]. For this study, we excluded subjects with end-stage renal disease prior to study enrollment (*n* = 43) or if they were missing SCr values on day 1 or 2 of enrollment (*n* = 42). AKI was defined as an increase in SCr of 0.3 mg/dl from a “baseline” SCr value or a decrease in urine output < 0.5 ml/kg/h over 24 h within the first 3 days of enrollment. We derived an approximation of the baseline SCr from the nadir measured over the 3-day period. This approach to defining AKI has been described previously [23–25]. AKI severity was determined using modified KDIGO criteria based on the maximal difference between the nadir creatinine and the maximal creatinine or the minimal urine output over the 3-day period.

AKI subphenotypes were defined as previously described [13]. The resolving subphenotype was defined by a decrease of 0.3 mg/dl or 25% in SCr from its maximum during the first 3 days of study enrollment. All subjects with AKI who did not meet this criterion were classified as having a nonresolving subphenotype [13]. Sepsis-2 was defined by the presence of a suspected infection in addition to SIRS. Sepsis-3 was defined by the presence of a suspected infection and a Sequential Organ Failure Assessment score of 2 points or more [26]. Septic shock was defined by the need for vasopressor therapy [26] in subjects with sepsis.

### Biomarker values

Blood for plasma biomarker measurements was collected during the first 24 h of study enrollment. The blood was collected in ethylenediaminetetraacetic acid (EDTA)-treated sterile tubes and centrifuged. Plasma was then aliquoted and frozen at −80 °C. The samples were stored for a variable number of years, but the plasma samples were freeze-thawed a maximum of one time prior to running the biomarker measurements. All biomarkers were measured on the same day using electrochemiluminescence immunoassays (Meso Scale Discovery, Rockville, MD, USA). The biomarkers were measured for research purposes. The biomarkers were run in singlets, and we used an EDTA plasma control sample on each plate to assess assay performance. Samples were diluted to fit within the dynamic range of each assay: IL-6, IL-8, and sTNFR-1 were diluted 0.08–2500 pg/ml; Ang-1 was diluted 3–100,000 pg/ml; Ang-2 was diluted 0.5–10,000 pg/ml; sVCAM-1 was diluted 0.05–1000 pg/ml; and sFas was diluted 40–5000 pg/ml. The samples that fell below the lower limit of detection or above the upper limit of detection were assigned those values. The number of samples below or above the limit of detection is provided in Additional file 1: Table S1. The intra-assay coefficients of variation ranged from 12 to 15 for the biomarkers. Additionally, we remeasured a random subset of these samples and analyzed the replicates using Pearson’s correlation. The averaged Pearson’s correlation was 0.95 with an SD of 0.06.

### Statistical analysis

For baseline characteristics, we report continuous variables as mean ± SD and categorical variables as number and percent. Approximately 6% or less of the study participants were missing data on APACHE III (<1%), body mass index (1.7%), and race (6%). For the regression analyses, data for participants with missing values for these covariates were imputed using chained equations and combined using Rubin’s rules [27]. No imputations were completed for exposure or outcome measures. Associations between AKI subphenotype and hospital mortality were identified using relative risk (RR) regression [28], given that hospital mortality in subjects with AKI was relatively common (i.e., > 15%). The final model was adjusted for age, sex, race, body mass index, diabetes mellitus, APACHE III score, vasopressor use, mechanical ventilation, and KDIGO stage of AKI [13]. The covariates were chosen a priori on the basis of biologic plausibility that they could confound the associations of biomarkers with AKI subphenotypes.

Plasma biomarker concentrations were tabulated by AKI status (no AKI, resolving and nonresolving) and reported as median and IQR. Biomarker levels were log_2_-transformed because they are known to be heavily right-skewed with a very wide range. A two-tailed *t* test was performed to evaluate the association of biomarkers with resolving versus nonresolving AKI subphenotypes.

Univariate and multivariate associations between biomarker concentrations and AKI subphenotype are presented as RRs per doubling of the biomarker concentration. We performed RR regression using a multivariate generalized linear model to test for associations between biomarker levels (independent variable) and AKI subphenotype (dependent variable). Gaussian model and robust SE estimates were used if the binomial function did not allow for model convergence. Variables to include in the model were decided a priori on the basis of biologic plausibility and prior literature [1, 2, 29, 30]. The first adjusted model included baseline age, diabetes mellitus, and body mass index. The second model added APACHE III scores, which were based on the maximum values during the first hospital day. Data are presented as RR and 95% CI. All analyses were performed using Stata release 13.1 software (StataCorp, College Station, TX, USA).

## Results

Detailed characteristics of the HMC-SIRS cohort have been published previously [22, 31]. The average age in the HMC-SIRS cohort was 54 years (SD ±16); 65% were men; 77% were Caucasian; 28% had a history of diabetes mellitus; and 9% had chronic kidney disease. Sepsis-3 was the admission diagnosis of 58% of subjects. During the first 3 days of ICU admission, 64% of subjects required mechanical ventilation for any period, and 23% required vasopressors (Table 1). Of 1241 eligible subjects, 868 subjects (70%) developed AKI within 3 days of study enrollment. The incidence of AKI is consistent with prior reports identifying the rate of AKI in ICU populations [32, 33]. Of the subjects with AKI, 502 subjects had a resolving subphenotype, and 366 had a nonresolving subphenotype. The proportion of subjects with a nonresolving subphenotype was greater than in our prior work identifying AKI subphenotypes in post-trauma and mixed medical-surgical ICU populations [13]. Subjects with the resolving and nonresolving subphenotypes had similar characteristics in multiple categories, including age, sex, body mass index, APACHE III score, cirrhosis, chronic kidney disease, need for vasopressors, and maximum SCr during the first 72 h of ICU care. Subjects with the nonresolving subphenotype had a higher rate of Sepsis-3 (66% versus 57%).

**Table 1:** Patient characteristics in Harborview Medical Center cohort with systemic inflammatory response syndrome

Clinical variables	No AKI	AKI		Total
		Resolving AKI	Nonresolving AKI	
Total	373	502	366	1241
Baseline demographics				
Age, years	53 ± 17	55 ± 15	55 ± 17	54 ± 16
Male sex, *n* (%)	233 (63)	323 (64)	250 (68)	806 (65)
Body mass index, kg/m^2^	29.4 ± 10.2	31.2 ± 16.5	32.0 ± 19.4	30.8 ± 15.6
Race, *n* (%)				
Caucasian	267 (77)	360 (76)	272 (79)	899 (77)
African American	41 (12)	66 (14)	29 (8)	136 (12)
Asian	26 (7)	35 (7)	29 (8)	90 (8)
Native American	15 (4)	13 (3)	14 (4)	42 (4)
Unknown	24 (6)	28 (6)	22 (6)	74 (6)
APACHE III (24 h)	38 ± 18	57 ± 27	55 ± 27	50 ± 26
Comorbidities, *n* (%)				
Diabetes mellitus	78 (21)	167 (33)	98 (27)	343 (28)
Cirrhosis	37 (10)	39 (8)	38 (10)	114 (9)
Chronic kidney disease	16 (4)	55 (11)	43 (12)	114 (9)
Congestive heart failure	17 (5)	58 (12)	24 (7)	99 (8)
Chronic obstructive lung disease	64 (17)	82 (16)	59 (16)	205 (17)
ICU events				
Mechanical ventilation (72 h)	178 (48)	354 (71)	261 (71)	793 (64)
Vasopressors (72 h)	39 (10)	140 (28)	108 (30)	287 (23)
Sepsis-2	227 (61)	348 (69)	268 (73)	843 (68)
Sepsis-3	190 (51)	287 (57)	242 (66)	719 (58)
Septic shock	34 (14)	122 (24)	90 (25)	246 (20)
Admission Scr	0.8 ± 0.4	1.9 ± 1.7	1.6 ± 1.9	1.5 ± 1.6
Maximum SCr (72 h)	0.8 ± 0.4	2.0 ± 1.8	2.2 ± 2.4	1.7 ± 1.8
KDIGO stage of AKI				
Stage 0	373 (100)	N/A	N/A	373 (30)
Stage 1	N/A	219 (44)	158 (43)	377 (30)
Stage 2	N/A	155 (31)	91 (25)	250 (20)
Stage 3	N/A	128 (26)	117 (32)	245 (20)

*Abbreviations: AKI* Acute kidney injury, *APACHE III* Acute Physiology and Chronic Health Evaluation III, *ICU* Intensive care unit, *KDIGO* Kidney Disease: Improving Global Outcomes, *SCr* Serum creatinine

Data are shown as mean ± SD, number of subjects (%), or median (IQR), as appropriate

Because we tested eight different associations between biomarker concentrations and a nonresolving AKI subphenotype, we completed a correlation matrix using a Spearman’s correlation test to evaluate collinearity between biomarkers. Other than correlations of 0.66 between sFas and sTNFR-1 and 0.64 between sTNFR-1 and Ang-2, the rest of the correlations between biomarkers were < 0.6 (Additional file 1: Table S2). Thus, we chose the most conservative estimate of a Bonferroni-corrected *p* value cutoff of 0.05/8 = 0.00625 to test for significance.

### AKI subphenotypes, risk of death, and need for renal replacement therapy

Consistent with our prior report, the nonresolving AKI subphenotype was associated with a greater risk of hospital mortality than in patients with no AKI after adjustment for multiple factors, including APACHE III score, mechanical ventilation, vasopressor use, and KDIGO stage of AKI (Table 2) (adjusted RR [model C] 2.9, 95% CI 1.3, 6.4), whereas the resolving AKI subphenotype showed no increased risk of hospital mortality after adjustment for the same variables (Table 2) (adjusted RR [model C] 1.4, 95% CI 0.6, 3.7). There was an increased risk of death with a nonresolving AKI subphenotype within each KDIGO stage of AKI (Additional file 1: Table S3). To account for potential misclassification of AKI, we completed sensitivity analyses to evaluate the risk of a nonresolving subphenotype in a population of patients with KDIGO stage 2 or 3 AKI. In this population with more severe AKI, the risk of death in the nonresolving subphenotype persisted (Additional file 1: Table S4). Of all subjects in the cohort, 91 (7%) required new initiation of renal replacement therapy (RRT) during their hospitalization. Of the subjects with a resolving subphenotype, 23 (5%) required RRT compared with 65 (21%) with a nonresolving subphenotype. The need for RRT in the resolving subphenotype group was subsequent to a new AKI event. The adjusted risk of requiring inpatient initiation of RRT was greater in a nonresolving AKI subphenotype than among patients with a resolving AKI subphenotype (RR 9.7, 95% CI 2.1, 44.4) (Additional file 1: Table S5).

**Table 2:** Risk for hospital mortality by Kidney Disease: Improving Global Outcomes stage and acute kidney injury subphenotype

			Relative risk (95% CI)			
	No. of patients	Deaths, *n* (%)	Unadjusted model	Adjusted model A	Adjusted model B	Adjusted model C
No AKI	373	11 (3)	1.00 (reference)			
KDIGO AKI stage						
Stage 1	377	51 (14)	4.5 (2.4, 8.6)	3.9 (1.9, 7.7)	2.2 (1.1, 4.6)	–
Stage 2	250	30 (12)	4.0 (2.1, 7.9)	3.3 (1.5, 7.0)	1.6 (0.7, 3.9)	–
Stage 3	245	51 (21)	7.0 (3.7, 13.1)	5.9 (3.0, 11.6)	1.9 (0.8, 4.4)	–
AKI subphenotype						
Resolving	502	57 (11)	3.9 (2.1, 7.2)	3.2 (1.5, 6.6)	1.3 (0.6, 3.1)	1.4 (0.6, 3.7)
Nonresolving	366	75 (21)	7.0 (3.8, 12.9)	5.7 (3.0, 11.2)	2.7 (1.3, 5.6)	2.9 (1.3, 6.4)

*AKI* Acute kidney injury, *KDIGO* Kidney Disease: Improving Global Outcomes

Adjustment variables were as follows:

Model A: age, sex, race

Model B: Model A + body mass index, diabetes mellitus, Acute Physiology and Chronic Health Evaluation III, vasopressor use, mechanical ventilation

Model C: Model B + KDIGO stage of AKI

### Biomarker levels and risk for AKI subphenotypes

**Fig. 1:**
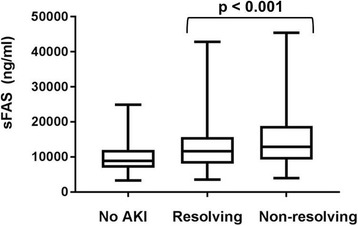
Soluble Fas (sFas) biomarker levels in the study cohort. Box plots showing median, interquartile range (*box*), and upper and lower adjacent values (*bars*) for biomarker levels, stratified by no acute kidney injury (AKI), resolving acute kidney injury, and nonresolving acute kidney injury. *p* Value is for comparison of resolving with nonresolving AKI

Among patients with AKI, univariate analysis showed that only sFas levels were significantly different between the resolving and nonresolving AKI subphenotypes after Bonferroni correction (Table 3). In multivariate analyses adjusting for potential confounders known to be associated with circulating biomarker levels and risk for AKI, including age, diabetes mellitus, body mass index, and APACHE III scores [1, 2, 34] (Table 4), we found that only sFas levels were associated with a nonresolving, as opposed to a resolving, AKI subphenotype (adjusted RR 1.16 per doubling of sFas levels, 95% CI 1.05, 1.28) after Bonferroni correction. Figure 1 shows the stepwise increase in sFas biomarker concentrations in those with no AKI, a resolving AKI subphenotype, and a nonresolving AKI subphenotype.

**Table 3:** Plasma biomarker concentrations by acute kidney injury subphenotype

Biomarker	No. of patients	Biomarker concentration, median (IQR)			
		No AKI	Resolving AKI	Nonresolving AKI	Resolving versus nonresolving (*p* value)
Endothelial dysfunction					
Ang-1, pg/ml	1212	6382 (3114, 10,409)	4393 (1957, 8856)	4033 (1638, 8048)	0.315
Ang-2, pg/ml	1221	7985 (4636, 14,996)	14,924 (8367, 29,425)	15,126 (7047, 35,138)	0.287
Ang-2/Ang-1	1212	1.3 (0.6, 3.5)	3.6 (1.1, 12.4)	3.6 (1.1, 18.1)	0.039
sVCAM-1, ng/ml	1222	481 (382, 687)	530 (388, 783)	571 (446, 842)	0.023
Apoptosis and inflammation					
sTNFR-1, pg/ml	1161	5380 (3961, 8000)	10,063 (6147, 15,566)	9838 (5765, 18,358)	0.010
sFas, pg/ml	1223	8810 (6880, 11,926)	11,586 (8095, 15,700)	12,879 (8938, 17,682)	0.001^a^
IL-6, pg/ml	1149	75 (31, 178)	137 (59, 351)	147 (58, 375)	0.536
IL-8, pg/ml	1160	11 (5, 20)	13 (7, 35)	14 (7, 33)	0.420

*Abbreviations: AKI* Acute kidney injury, *Ang-1* Angiopoietin 1, *Ang-2* Angiopoietin 2, *IL* Interleukin, *sFas* Soluble Fas, *sTNFR-1* Soluble tumor necrosis factor receptor 1, *sVCAM-1* Soluble vascular cell adhesion molecule 1

^a^ *p* < 0.00625 based on Bonferroni correction for multiple hypotheses

**Table 4:** Associations between biomarker levels and risk of nonresolving acute kidney injury subphenotype

Biomarkers	Unadjusted RR^a^ (95% CI)	*p* Value	Adjusted^b^ model A, RR (95% CI)	*p* Value	Adjusted model B, RR (95% CI)	*p* Value
Endothelial dysfunction						
Ang-1	0.96 (0.91, 1.00)	0.068	0.96 (0.91, 1.00)	0.073	0.95 (0.91, 1.00)	0.049
Ang-2	1.00 (0.95, 1.07)	0.850	0.99 (0.94, 1.06)	0.851	1.00 (0.94, 1.07)	0.923
Ang-2/Ang-1	1.04 (1.00, 1.08)	0.029	1.02 (0.98, 1.05)	0.291	1.03 (1.00, 1.06)	0.160
sVCAM-1	1.12 (1.03, 1.22)	0.007	1.11 (1.02, 1.21)	0.017	1.11 (1.02, 1.21)	0.016
Apoptosis and inflammation						
IL-6	1.00 (0.97, 1.05)	0.604	1.00 (0.96, 1.04)	0.977	1.00 (0.97, 1.04)	0.830
IL-8	1.01 (0.97, 1.05)	0.718	1.00 (0.97, 1.04)	0.781	1.00 (0.97, 1,05)	0.676
sFas	1.21 (1.16, 1.28)	0.001^c^	1.14 (1.12, 1.26)	0.001^c^	1.16 (1.05, 1.28)	0.005^c^
sTNFR-1	1.06 (0.98, 1.15)	0.144	1.04 (0.96, 1.13)	0.301	1.05 (0.97, 1.14)	0.235

*Abbreviations: Ang-1* Angiopoietin 1, *Ang-2* Angiopoietin 2, *IL* Interleukin, *RR* Relative risk, *sFas* Soluble Fas, *sTNFR-1* Soluble tumor necrosis factor receptor 1, *sVCAM-1* Soluble vascular cell adhesion molecule 1

^a^Relative risks presented per doubling of each biomarker

^b^Adjustment variables were as follows:

Model A: age, diabetes mellitus, body mass index

Model B: model A + Acute Physiology and Chronic Health Evaluation III

^c^ *p* < 0.00625 based on Bonferroni correction for multiple hypotheses

### Subgroup analysis in septic shock

A greater percentage of patients in the nonresolving AKI subphenotype had septic shock (sepsis and requirement for vasopressor therapy during the first 72 h of ICU admission), potentially confounding our analyses. To minimize this possibility, we examined the subgroup of patients with septic shock (*n* = 205). In this subgroup, 34 (17%) had no AKI, 122 (60%) had a resolving subphenotype, and 90 (44%) had a nonresolving subphenotype. sFas continued to be strongly associated with a nonresolving subphenotype (RR 1.41, 95% CI 1.12, 1.80, *p* = 0.004). Of note, when we assessed for associations between biomarker levels and AKI subphenotype in the subgroup with septic shock, we found that, in addition to sFas, biomarkers of endothelial dysfunction were associated with AKI subphenotypes. Higher soluble VCAM (RR 1.29, 95% CI 1.08, 1.54, *p* = 0.005) and lower Ang-1 (RR 0.84, 95% CI 0.78, 0.89, *p* < 0.001) were associated with the nonresolving AKI subphenotype (Additional file 1: Table S6).

## Discussion

In our analysis of a large cohort of critically ill subjects, we confirmed the presence of two AKI subphenotypes based on the trajectory of SCr in the first 3 days of ICU admission. As we previously demonstrated, subjects with a resolving AKI subphenotype have a similar risk of mortality and RRT as that of subjects with no AKI, but subjects with a nonresolving SCr trajectory have a twofold higher risk of death [13]. In contrast to a recently published work in which researchers excluded subjects with KDIGO stage 1 AKI to identify trajectories of AKI, we included all subjects with AKI in our analyses [11]. Minor changes in SCr are important [35], and KDIGO stage 1 AKI includes a large, heterogeneous population of all subjects with AKI (approximately 43% of subjects with AKI in our study were in KDIGO stage 1). To evaluate the pathophysiology of these distinct AKI subphenotypes, we measured plasma biomarkers associated with the development of AKI in key biologic pathways: inflammation, apoptosis, and endothelial dysfunction. We found that higher levels of sFas were associated with an elevated risk of developing a nonresolving AKI subphenotype.

Fas is a type 1 membrane protein that belongs to the tumor necrosis factor receptor 4 superfamily, which activates intracellular signaling after binding of Fas ligand (FasL) [36]. Fas ligation leads to a series of intracellular signaling events, culminating in activation of the death-inducing signaling complexes, which promote the activation of caspase-8-mediated apoptosis. Additionally, Fas ligation is believed to have an inflammatory role through cytokine production and then recruitment of proinflammatory cells [37]. sFas is a truncated form of Fas believed to result from proteolytic cleavage of membrane-bound receptors or alternative splicing of messenger RNA transcripts [38]. We have previously shown that genetic polymorphisms in *FAS*-related genes are associated with the development of AKI in subjects with acute respiratory distress syndrome (ARDS) [39]. Other studies have implicated the Fas pathway in the development of AKI in non-ARDS populations, such as patients with infection and chronic kidney disease [40–42]. Moreover, Ko et al. [14] showed in a murine model that a genetic deficiency of functional FasL protects mice from bilateral renal ischemia-reperfusion injury as measured by decreased apoptosis based on caspase 3 immunohistochemical staining, as well as decreases in SCr. Further, these authors also showed that pharmacologic blockade of FasL with an anti-FasL monoclonal immunoglobulin G antibody protected the kidneys of wild-type mice from ischemia-reperfusion injury.

It is well known that septic shock is a strong risk factor for AKI in the critically ill. We found a higher prevalence of sepsis and vasopressor use in the nonresolving AKI subphenotype than in the resolving AKI subphenotype. To account for the confounding of septic shock on the development of a nonresolving AKI subphenotype, we completed a sensitivity analysis limited to subjects with septic shock. We found that the association of sFas with increased risk of a nonresolving AKI subphenotype was unchanged. Of note, biomarker levels of endothelial dysfunction (Ang-1 and sVCAM) were also associated with risk for nonresolving AKI in the septic shock subset. These findings suggest that sFas is linked to the nonresolving AKI subphenotype, not just to the severity of sepsis, and that endothelial dysfunction may play a larger role in the development of AKI in patients with septic shock than in the overall AKI population.

The identification of distinct molecular profiles for different AKI subphenotypes has several potential implications. First, our findings suggest that inclusion of subjects with both resolving and nonresolving AKI subphenotypes in clinical trials of treatments for AKI adds a degree of disease heterogeneity that could reduce the efficacy of specific therapeutic interventions based on molecular mechanisms [43]. Second, our findings suggest that the Fas/FasL system may be differentially involved in the development of a nonresolving AKI subphenotype. We do not yet know whether the increased circulating levels of sFas are simply associated with or causal in the development of AKI. However, we do know from animal models that deficiency or blockade of the Fas/FasL system can protect from renal injury [14]. Thus, we favor a model in which increased sFas represents a byproduct of increased Fas pathway activity. Future studies will address the mechanistic role of sFas in the development of AKI. Third, our findings of associations between markers of endothelial dysfunction and the nonresolving AKI subphenotype in patients with septic shock support prior work demonstrating the protective effect of Ang1/Tie2 agonists in animal models of organ dysfunction in sepsis [44], and they suggest that targeting this pathway may have more of an impact in subjects with septic shock at risk for the nonresolving AKI subphenotype. Fourth, the identification of molecular subphenotypes in alternative heterogeneous diseases, such as asthma and lung cancer, has informed novel treatment strategies [45, 46]. Similarly, continued molecular characterization of AKI subphenotypes may identify therapeutic pathways for further investigation.

This study has several important strengths that support its novelty and robustness. First, to the best of our knowledge, this is the first study to link sFas concentrations and the development of AKI. Second, our use of a large, prospective ICU cohort allowed for more precise assessments of the associations between biomarkers and risk for AKI. Indeed, this is one of the largest published studies of biomarkers and risk for AKI. Third, we minimized the potential for type I error by using the Bonferroni correction to account for multiple hypothesis testing. Fourth, the simultaneous evaluation of two distinct pathways implicated in the pathogenesis of AKI allowed for a comparison of the relative strengths of association. Fifth, the association of sFas concentrations with a nonresolving subphenotype persisted in the subgroup of patients with severe AKI (KDIGO stage 2 or 3).

The study has several important limitations. First, our definition of “resolving” AKI required an SCr decrease of only 0.3 mg/dl or 25% from the maximum value. This relatively minimal decrease raises the possibility that a significant number of patients who actually had a nonresolving AKI subphenotype were misclassified as having a resolving AKI subphenotype. However, we have previously shown that this definition is superior to alternative definitions of the subphenotypes with regard to the separation by outcome (mortality) [13]. Even when stratified by KDIGO AKI stage, the patients with the nonresolving AKI subphenotype had a higher mortality and greater need for RRT than those with the resolving subphenotype. Also, misclassification of this type would be expected to add experimental noise and to have biased our results toward the null. Second, our study does not provide insight into the functional significance of elevated sFas in AKI. Future studies are needed to evaluate the relationship of sFas to sFasL and to address mechanistic questions of the role of sFas in AKI. Third, it is unknown if sFas is filtered from the glomerular capillary. In one prior study, researchers reported that sFas concentrations increased with worsening kidney function [47], but it is unknown if this increase was due to activation of the Fas/FasL system, entirely a function of decreased filtration of circulating sFas, or a combination of both. Fourth, clinical factors besides AKI, such as sepsis [48], major trauma [49], or active malignancy [50–52], have been associated with increased circulating levels of sFas. To account for these additional clinical factors, we excluded from our study patients with major trauma or active malignancy. Additionally, we completed a sensitivity analysis of patients with septic shock to determine if AKI influences sFas levels independently of sepsis.

## Conclusions

We have shown that a biomarker of Fas pathway activity, sFas, is associated with the risk of developing a nonresolving AKI subphenotype in critically ill patients without major trauma, severe immune suppression, or active cancer. In contrast, biomarkers of endothelial dysfunction demonstrated an association with this subphenotype only in the subgroup of subjects with septic shock. These findings extend experimental data from animal models, suggesting that activation of the Fas pathway and, to a lesser extent, suppression of the Ang-1 axis play an important role in the pathogenesis of AKI. The continued molecular identification of AKI subphenotypes may allow recognition of subjects at high risk for poor outcomes, might facilitate the identification of novel therapeutic targets, and might allow for targeted enrollment in clinical trials.

## Additional files

Additional file 1: Supplemental data file that includes supplementary tables referenced in the text. (DOCX 32 kb)

## Abbreviations

AKI: Acute kidney injury
Ang-1: Angiopoietin 1
Ang-2: Angiopoietin 2
APACHE III: Acute Physiology and Chronic Health Evaluation III
ARDS: Acute respiratory distress syndrome
EDTA: Ethylenediaminetetraacetic acid
FasL: Fas ligand
HMC-SIRS: Harborview Medical Center cohort with systemic inflammatory response syndrome
ICU: Intensive care unit
IL: Interleukin
KDIGO: Kidney Disease: Improving Global Outcomes
RR: Relative risk
RRT: Renal replacement therapy
SCr: Serum creatinine
sFas: Soluble Fas
SIRS: Systemic inflammatory response syndrome
sTNFR-1: Soluble tumor necrosis factor receptor 1
sVCAM: Soluble vascular cell adhesion molecule
