# Supplementary material for: Circulating levels of soluble Fas (sCD95) are associated with risk for development of a nonresolving acute kidney injury subphenotype
Source: Crit Care. 2017 Aug 17;21:217. doi: 10.1186/s13054-017-1807-x (PMC5559814; doi:10.1186/s13054-017-1807-x)
Supplement: Supplementary file 1 — Supplemental data file that includes supplementary tables referenced in the text. (DOCX 32 kb) [file 13054_2017_1807_MOESM1_ESM.docx]

**ONLINE SUPPLEMENATRY MATERIALS**

**Title:** Circulating levels of soluble Fas (sCD95) are associated with risk for development of a non-resolving acute kidney injury sub-phenotype

**Authors:** Pavan K. Bhatraju, MD MSc, Cassianne Robinson-Cohen, PhD, Carmen Mikacenic, MD,

Susanna Harju-Baker, PhD, Victoria Dmyterko, BS, Natalie S. J. Slivinski, MS, W. Conrad Liles, MD PhD, Jonathan Himmelfarb, MD, Susan R. Heckbert, MD PhD, Mark M. Wurfel, MD PhD

**Table S1.** Number of subjects above or below the limit of detection for each biomarker

| Biomarker | Below LLOD* (n) | Above ULOD** (n) |
| --- | --- | --- |
| IL-6 | 0 | 33 |
| IL-8 | 0 | 5 |
| sFas | 0 | 0 |
| sTNFR-1 | 2 | 23 |
| Ang1 | 0 | 0 |
| Ang2 | 0 | 59 |

*LLOD – lower limit of detection

**ULOD – upper limit of detection

**Table S2. Correlation Matrix of Biomarkers**

|  | **sFas** | **sTNFR-1** | **IL-6** | **IL-8** | **Ang-2** | **Ang-1** | **Ang-2/Ang-1 Ratio** | **sVCAM** |
| --- | --- | --- | --- | --- | --- | --- | --- | --- |
| **sFas** | 1.00 |  |  |  |  |  |  |  |
| **sTNFR-1** | 0.66 | 1.00 |  |  |  |  |  |  |
| **IL-6** | 0.15 | 0.53 | 1.00 |  |  |  |  |  |
| **IL-8** | 0.34 | 0.46 | 0.40 | 1.00 |  |  |  |  |
| **Ang-2** | 0.47 | 0.64 | 0.47 | 0.35 | 1.00 |  |  |  |
| **Ang-1** | -0.31 | -0.28 | -0.18 | -0.02 | -0.32 | 1.00 |  |  |
| **Ang2/Ang1 Ratio** | 0.47 | 0.55 | 0.39 | 0.21 | 0.79 | -0.81 | 1.00 |  |
| **sVCAM** | 0.59 | 0.52 | 0.22 | 0.35 | 0.45 | -0.33 | 0.47 | 1.00 |

**r_s_ values determined by Spearman Correlation Coefficients**

**Table S3. Mortality by KDIGO Stage and AKI Sub-phenotype**

|  | | **KDIGO Stage of AKI** | | |
| --- | --- | --- | --- | --- |
|  |  | Stage 1 (n=377) | Stage 2 (n=250) | Stage 3 (n=245) |
| **AKI Sub-phenotypes** | Resolving (n=502) | 25/219 (11%) | 17/155 (11%) | 15/128 (12%) |
|  | Non-Resolving (n=366) | 26/158 (16%) | 13/91 (14%) | 36/117 (31%) |

**Table S4. Risk for Hospital Mortality by KDIGO stage and AKI Sub-phenotype in patients with KDIGO Stage 2 and 3 AKI**

|  |  |  |  |  |  |
| --- | --- | --- | --- | --- | --- |
|  | **n** | **Deaths**  **n (%)** | **Unadjusted Model** | **Adjusted Model a** | **Adjusted Model b** |
|  |  |  |  |  |  |
| **No AKI** | 373 | 11 (3) | 1.00 (ref) |  |  |
| **KDIGO AKI Stage** |  |  |  |  |  |
| Stage 2+3 | 377 | 81 (16) | 5.5 (3.0, 10.2) | 5.2 (2.6, 10.5) | 2.4 (1.0, 5.6) |
|  |  |  |  |  |  |
| **AKI Sub-phenotype** |  |  |  |  |  |
| Resolving | 283 | 32 (11) | 3.8 (2.0, 7.5) | 3.4 (1.5, 7.5) | 1.4 (0.5, 4.1) |
| Non-Resolving | 208 | 49 (24) | 8.0 (4.2, 15.0) | 7.3 (3.7, 14.4) | 3.4 (1.5, 7.7) |

Adjustment Variables

Model a. Age, Gender, Race

Model b. Model a + Body Mass Index, Diabetes Mellitus, APACHE III, Vasopressor Use, Mechanical Ventilation

**Table S5. Risk for Renal Replacement Therapy by KDIGO stage and AKI Sub-phenotype**

|  |  |  |  |  |  |
| --- | --- | --- | --- | --- | --- |
|  | **n** | **RRT n (%)** | **Unadjusted Model** | **Adjusted Model a** | **Adjusted Model b** |
|  |  |  |  |  |  |
| **No AKI** | 373 | 2 (1) | 1.00 (ref) | 1.00 (ref) | 1.00 (ref) |
|  |  |  |  |  |  |
| **AKI Sub-phenotype** |  |  |  |  |  |
| Resolving | 492 | 23 (5) | 8.5 (2.0, 36.0) | 8.9 (1.9, 41.1) | 2.9 (0.6, 14) |
| Non-Resolving | 366 | 66 (18) | 33.6 (8.3, 136.4) | 30.4 (7.1, 130.1) | 9.7 (2.1, 44.4) |

Adjustment Variables

Model a. Age, Gender, Race

Model b. Model a + Body Mass Index, Diabetes Mellitus, APACHE III, Vasopressor Use, Mechanical Ventilation

**Table S6. Associations of biomarkers (per doubling of each biomarker) with non-resolving AKI sub-phenotype versus resolving AKI, in 205 patients with AKI and septic shock**

| **Biomarkers** | **Unadjusted RR (95%CI)** | **p** |  | **Adjusted Model a (95%CI)** | **p** |  | **Adjusted Model b RR (95%CI)** | **p** |
| --- | --- | --- | --- | --- | --- | --- | --- | --- |
| ***Endothelial Dysfunction*** |  |  |  |  |  |  |  |  |
| Ang-1 | 0.84 (0.79, 0.89) | <0.001* |  | 0.84 (0.78, 0.89) | <0.001* |  | 0.84 (0.78, 0.89) | <0.001* |
| Ang-2 | 1.05 (0.93, 1.20) | 0.430 |  | 1.03 (0.90, 1.17) | 0.689 |  | 1.03 (0.90, 1.18) | 0.657 |
| Ang-2/Ang-1 | 1.11 (1.04, 1.17) | 0.001* |  | 1.10 (1.04, 1.17) | 0.002* |  | 1.10 (1.04, 1.17) | 0.001* |
| sVCAM-1 | 1.31 (1.11, 1.54) | 0.001* |  | 1.29 (1.08, 1.54) | 0.005* |  | 1.29 (1.08, 1.54) | 0.005* |
| ***Apoptosis and Inflammation*** |  |  |  |  |  |  |  |  |
| IL-6 | 1.00 (0.94, 1.07) | 0.942 |  | 0.98 (0.91, 1.05) | 0.544 |  | 0.98 (0.91, 1.06) | 0.616 |
| IL-8 | 0.96 (0.89, 1.03) | 0.235 |  | 0.94 (0.87, 1.02) | 0.162 |  | 0.95 (0.87, 1.03) | 0.205 |
| sFAS | 1.39 (1.11, 1.75) | 0.004* |  | 1.40 (1.11, 1.77) | 0.004* |  | 1.41 (1.12, 1.80) | 0.004* |
| sTNFR-1 | 1.27 (1.04, 1.54) | 0.019 |  | 1.23 (1.00, 1.51) | 0.043 |  | 1.24 (1.02, 1.52) | 0.035 |

Relative risks presented per doubling of each biomarker.

*p value < .00625 based on Bonferroni correction for multiple hypotheses

Adjustment Variables

Model a. age, DM, BMI

Model b. model a + APACHE III
